# Circulating LncRNAs landscape as potential biomarkers in breast cancer

**DOI:** 10.1002/cnr2.1722

**Published:** 2022-10-23

**Authors:** Zahra Pourramezan, Fatemeh Akhavan Attar, Maryam Yusefpour, Masoumeh Azizi, Mana Oloomi

**Affiliations:** ^1^ Department of Molecular Biology Pasteur Institute of Iran Tehran Iran; ^2^ Department of Molecular Medicine Biotechnology Research Center, Pasteur Institute of Iran Tehran Iran

**Keywords:** breast cancer detection, long non‐coding RNAs, real‐time PCR, whole blood samples

## Abstract

**Background:**

In Iran, the delay in diagnosis and treatment of breast cancer results in low survival rates.

**Aim:**

It is essential to characterize new therapeutic targets and prognostic breast cancer biomarkers. The rising evidence suggested that long non‐coding RNAs (lncRNAs) expression levels are deregulated in human cancers and can use as biomarkers for the rapid diagnosis of breast cancer.

**Methods:**

In the present study, a quantitative real‐time polymerase chain reaction (qRT‐PCR) technique was used to measure 20 oncogenic and tumor suppressor lncRNAs expression levels in whole blood samples of female breast cancer patients and healthy women. Receiver operating characteristic curve (ROC) was used to assess the diagnostic value of each selected lncRNA as a biomarker.

**Results:**

The results revealed that some circulating lncRNAs (MEG3, NBAT1, NKILA, GAS5, EPB41L4A‐AS2, Z38, and BC040587) were significantly down‐regulated in breast cancer patients compared to healthy women. In contrast, other circulating lncRNAs (H19, SPRY4‐IT1, XIST, UCA1, AC026904.1, CCAT1, CCAT2, ITGB2‐AS, and AK058003) were significantly up‐regulated in breast cancer patients compared to controls. It was shown that the expression levels of NKILA, and NBAT1 lncRNAs were related to tumor size, and BC040587 expression level related to age, node metastasis, tumor size, and grade (*p* < .05). The association between H19 and SPRY4‐IT1 lncRNAs with HER‐2 was confirmed statistically (*p* < .05). ROC curves illustrated that the blood levels of SPRY4‐IT1, XIST, and H19 lncRNAs have excellent potential in discriminating breast cancer from the healthy controls, showing an AUC of 1.0 (95% CI 1.0–1.0, *p* = .00), 0.898 (95% CI 0.815–0.981, *p* = .00), and 0.848 (95% CI 0.701–0.995, *p* = .01), respectively.

**Conclusion:**

In conclusion, the expression levels of circulating H19 and SPRY4‐IT1 lncRNAs in breast cancer patients could consider as the prognostic biomarkers and therapeutic targets in breast cancer, because of their excellent power in discriminating breast cancer from healthy individuals and the significant correlation of H19, and SPRY4‐IT1 lncRNAs with clinicopathological traits. We also suggest the possible application of BC040587 lncRNA as a diagnostic and prognostic indicator to assess tumor progression in case of verification in larger patients' cohorts.

## 1 INTRODUCTION

Breast cancer is the most common malignant disease, affecting about 2 million women worldwide in 2018.^1^ In Iran, breast cancer is the first leading cause of cancer death in females, including 27% of all cancers with an age‐standardized rate (ASR) 31 per 100 000. According to the latest statistics in Iran, 13 776 new breast malignancies are identified in 2018.^2^ The high level of breast cancer mortality is due to a lack of diagnostic markers for early detection, mammography screening programs, and suitable molecular markers for targeted and effective treatment opportunities. Late diagnosis may lead to cancer metastasis with less than 25% in 5‐year survival.^3^

Breast cancer lacks biomarkers with high specificity and sensitivity for general screening. Therefore, it is essential to search for novel biomarkers. Recently, the circulating lncRNA levels in cancer patients were nominated as a potential biomarker.^4^ Growing evidence has shown that lncRNA expression levels are deregulated in human cancers. Therefore, there is a possibility of using lncRNAs as therapeutic targets or potential biomarkers for the rapid diagnosis of breast cancer.^5^, ^6^, ^7^ Besides, evaluation of circulating lncRNA levels in body fluids can be considered as a noninvasive diagnostic biomarker for some cancers.^8^

Previous studies have reported that some oncogenic lncRNAs are overexpressed in various types of cancer and can serve as a prognostic marker. Overexpression of colon cancer‐associated transcript‐1 (CCAT1**)** indicated its role in malignancies' pathogenesis.^9^, ^10^, ^11^ Similarly, the oncogenic role of some lncRNAs confirmed by illustrating their up‐regulation in breast cancer tissues compared to adjacent normal tissues.^12^, ^13^, ^14^, ^15^, ^16^, ^17^ AC026904.1, Urothelial Carcinoma‐associated 1 (UCA1), SPRY4 intronic transcript 1 (SPRY4‐IT1), microvascular invasion in hepatocellular carcinoma (MVIH), Colon Cancer Associated Transcript 2 (CCAT2), promoter of CDKN1A antisense DNA damage activated RNA (PANDAR) and zinc finger antisense 1 (ZFAS1) and H19 lncRNA, is overexpressed in 73% of breast cancer tissues compared to healthy tissues.^18^ Besides, a series of experiments showed that knockdown of Z38 significantly inhibited tumor growth in breast cancer.^19^ We hypothesized that the mentioned lncRNAs might be up‐regulated in breast cancer and act as an oncogene.

According to the previous studies, some lncRNAs function as tumor suppressor genes, and their down‐regulation may proceed to invasion and metastasis and lessen the effectiveness of chemotherapeutic treatment. Neuroblastoma associated transcript‐1 (NBAT1) behaved as a tumor‐suppressor and down regulated in invasive breast cancer.^20^ Likewise, decreased expression levels of FGF14 antisense RNA 2 (FGF14‐AS2), X inactive specific transcript (XIST), BC040587, and MEG3 in breast cancer tissue and cell lines compared with corresponding normal control were associated with unfavorable survival in breast cancer.^21^, ^22^, ^23^ Meanwhile, down‐regulation of lncRNAs such as NF‐κB interacting lncRNA (NKILA), EPB41L4A antisense RNA 2 (EPB41L4A‐AS2), and Growth arrest‐specific transcript 5 (GAS5) inhibit breast cancer progression and may advance invasion and metastasis of breast cancer.^16^, ^24^, ^25^ There is controversy regarding down‐regulation or up‐regulation of lncRNA‐AK058003 in literatures.^26^, ^27^

There is a crucial necessity to develop an early detection platform in order to increase the breast cancer survivals. In addition, advances in breast cancer research will result in the novel diagnosis and treatment of breast cancer. There are few published reports of lncRNAs expression levels in breast cancer patients' blood. Therefore, in the current study, the expression profiles of 20 lncRNAs (H19, CCAT1, CCAT2, UCA1, SPRY4‐IT1, AK058003, Z38, MVIH, XIST, PANDAR, GAS5, ITGB2‐AS1, MEG3, AC026904.1, ZFAS1, NKILA, EPB41L4A‐AS2, FGF14‐AS2, NBAT1, BC040587) in blood samples of breast cancer patients and healthy females investigated by qRT‐PCR. Then, the association of lncRNAs expression profiles with clinicopathological features of breast cancer patients were assessed. Receiver operating curve (ROC) curve was used to assess the diagnostic value of each selected lncRNA as a biomarker and the correlation between the circulating levels of 20 lncRNAs were analyzed by Pearson correlation in the breast cancer patients.

## 2 METHODS

### 2.1 Patient and control specimens

Blood samples of 30 breast cancer patients were collected from different hospitals (Sina, Farmaniyeh, Moheb Kosar) in other parts of Tehran, Iran from, April to August 2019. All the clinicopathological features of samples were obtained from the medical records. None of the patients had undergone any preoperative cancer treatments, including radiotherapy or chemotherapy. Thirty blood samples of healthy women were collected with an average age of 40 years. We use “healthy normal” as the absence of any apparent disease as defined by Aagaard et al.^27^ We first screened volunteer blood donors using criteria based on health history, including the absence of systemic diseases such as cancer, hypertension, diabetes, and autoimmune disorders or immunodeficiency. Exclusion criteria for both groups included a body mass index (BMI) outside the range of 18.5–24.9 kg/m^2^, being pregnant, consuming alcohol, suffering from infectious diseases, and specified chronic diseases. Furthermore, we excluded individuals under certain medical treatments such as corticosteroids, immunosuppressive agents, antibiotics, or probiotics within the last 6 months.

Fresh blood was quickly transferred on ice to the Pasteur Institute of Iran. The Pasteur Institute committee (Ethical approval IR.PII.REC.1397.008) confirmed all experiments in accordance with the relevant guidelines and regulations. Methods were performed in accordance with relevant guidelines/regulations, and confirming that informed consent was obtained from all participants.

### 2.2 RNA isolation and cDNA synthesis

Total RNAs were extracted from blood samples using the Jena Bioscience kit (Germany) according to the manufacturer's instructions. Only samples with an A260:A280 ratio between 1.8 and 2.1 was considered for further analysis recorded by the microplate reader (BioTek, USA). The cDNA was synthesized using the BIO FACT kit according to the manufacturer's protocol. Briefly, 2 μg total RNA, 1 μg Oligodt, 10 μl Mastermix, and 7 μl RNase Free dH_2_O, were combined in a total reaction volume of 20 μl and incubated at room temperature for 5 min, followed by 50°C for 30 min.^15^

### 2.3 Quantitative real‐time PCR

The used primers in this study were shown in Table 1. The primers some used for the first time and are mentioned in the table and designed by AllelID, Gene Runner, Baecon designer, and Primer 3 software. The designed primers were finally checked by using the Beacon designer or Primer‐Blast on the NCBI website.

**TABLE 1:** Primers used for lncRNAs expression levels in breast cancer

Name	Location	Tumor suppressor/oncogene	Function	Primer sequence	References
H19	11p15.5	Oncogene	miRNA sponge miRNA precursor	GAGCCGATTCCTGAGTC GCCTTCCTGAACACCTTA	In this study
XIST	Inactive X‐chromosome		X‐chromosome silencing and cell growth	CTCCAGATAGCTGGCAACC AGCTCCTCGGACAGCTGTAA	28
GAS5	1q25.1	Tumor suppressor	Interaction with the mTOR pathway	CAAGCCGACTCTCCATACCT CTTGCCTGGACCAGCTTAAT	16
PANDAR	~5 kb upstream of CDKN1A		Regulation of G1/S transition	GTGGCCAAAGGATCTGACGA TCCCAACAAACAAGGGGTGG	17
CCAT1	8q24.21	Oncogene	miRNA sponge	TCATGTCTCGGCACCTTTCC TCATTACCAGCTGCCGTGTT	29
CCAT2	8q24.21	Oncogene	Regulation of Wnt/catenin signaling pathway	TCATGTCTCGGCACCTTTCC AAGAGGGAGGTATCAACAGAGAC	In this study
UCA1	19p13.12	Oncogene	microRNA sponge; regulation of KLF4‐KRT6/13 signaling pathway and Metastasis	TTGTCCCCATTTTCCATCAT TTTGCCAGCCTCAGCTTAAT	30
BC040587	3q13.31	Tumor suppressor	Unknown	AATGACTTCACAGCAAGG GAGATGCTGCTGGTGAGTAG	In this study
SPRY4‐IT1	Chromosome 5	Oncogene	Promote cell proliferation, increase invasion and metastasis, inhibit apoptosis, advanced clinical stage, poor prognosis	CGATGTAGGATTCCTTTCA AGCCACATAAATTCAGCAGA	31
NBAT1	6p22.3	Tumor suppressor	Mediating transcriptional silencing	TCAGCAGAAACGGCACGAT	20
				AGATGACCCAGGCACCTCC	
AK058003	10q22	Oncogene	Regulating ‐synuclein gene (SNCG) expression	ACTGGTTCATAGTTAGGCTGGAT GGGAACAAAGATGGTTTCTACGT	26
Z38	3q11.2	Oncogene	Unknown	AGGTAAAAGGAACTGGCAACGC AGTGGGATTGTGGAGACGGTGT	32
FGF14‐AS2	13q33.1	Tumor suppressor	Unknown	AGGTTCATAGTTGCCAGAC AGTTCCAGTTACCATCTTCA	21
MVIH	10q22	Oncogene	Unknown	AGCACTTTGGAAGGCTTAGACA GAGACAGGATTTAGCCGTGTTG	33
EPB41L4A‐AS2	5p22.2	Tumor suppressor	Unknown	TCAAAACTACGTCTGATGCCAAA CGGAGCAGGTGCAATCTGT	25
NKILA	20q13	Tumor suppressor	Suppressing NF‐κB activation and EMT	ACCACTAAGTCAATCCCAGGTG AACCAAACCTACCCACAACG	34
ZFAS1	20q13.13	Tumor suppressor		CCAGTGGTGACTCCCTCTTCCAAAGAG GTTCAGGAAGCCATTCGTTCT	35
AC026904.1	8q11.21			GACTTAGGACCACTTAGCA CCACGATACCCACTTCTT	In this study
MEG3	14q32.2	Tumor suppressor	Proliferation and EMT	CTGGCATAGAGGAGGTGA TGGAGGTGAGGAAGGAAG	In this study
ITGB2‐AS1	10p11.22	Oncogene	Migration and Invasion	TTAGTGGTCTGCGAAGGTG AGGAGATGGAACGAGGAAA	36

The expression levels of lncRNAs were quantified by Eva Green premix (WisPure qPCR Master). A 2 μl cDNA, 10 μl Master‐mix, 6 μl water, and 1 μl of each primer (Metabion, Germany) were used for qPCR. The real‐time PCR conditions were as follows: 95°C for 10 min, 40 cycles of 95°C for 10 s, 60°C for 15 s, and 72°C for 20 s, which was done by Rotor‐Gene Q (Corbett, Germany). All experiments were performed in double, and LinReg PCR software (version 2014) was used to calculate each chart's Ct amount. The REST program (2009 software) was used to calculate Fold changes. The Heatmap depiction of lncRNAs expression levels (columns) of patients compared with healthy normal women was illustrated using http://www.heatmapper.ca/website.

### 2.4 Statistical analysis

The statistical program for SPSS 18.0 (SPSS, Chicago, IL, USA) was employed to analyze all the data. Data are expressed as the mean ± standard deviation. For comparisons between two groups, the Student's *t*‐test was used while one‐way analysis (ANOVA) and Bonferroni post hoc test were used to compare multiple groups. The *χ*2 test was applied to analyze the association between lncRNAs expression and clinicopathological status. We categorize the lncRNA expression into high/low groups and clinopathological traits to different groups (according to Table 2) before performing chi‐square tests. The ROC analysis was performed to calculate the area under the ROC curve (AUC) detect the cut‐off values and to evaluate the diagnostic efficacy of the different examined biomarkers. The optimal cut‐point value defines as the point minimizing the summation of absolute values of the differences between AUC and sensitivity and AUC and specificity provided that the difference between sensitivity and specificity is minimum.^37^ The correlation analysis between expression levels of 20 lncRNAs in the blood samples of breast cancer patients were done by Pearson correlation. *p* Value <.05 was considered to indicate a statistically significant difference in all cases.

**TABLE 2:** Clinicopathological features of 30 Iranian healthy women and 30 breast cancer patients

Clinicopathological features	Frequency
*Healthy women*	
Age	
≤50	17
<50	13
*Cancer patients*	
Age	
≤50	16
<50	14
Tumor size (cm)	
<2.5	18
≤2.5	12
Differentiation grade	
G1/G2	19
G3	11
Lymph node metastasis	
Positive	14
Negative	16
TNM staging (tumor, node, metastases)	
I	14
II	7
III	9
Histological type	
IDC	28
ILC	2
Estrogen receptor (ER)	
Positive	10
Negative	17
Unknown	3
Progesterone receptor (PR)	
Positive	10
Negative	17
Unknown	3
HER2 statues	
Positive	3
Negative	24
Unknown	3

## 3 RESULTS

### 3.1 Clinical characteristics of the study population

Characteristics of all study population were presented in Table 2. The median age of patients was 50 years. The age in breast cancer patients was higher than in healthy women (*p* < .001).

### 3.2 Expression levels of circulating lncRNAs

**FIGURE 1:**
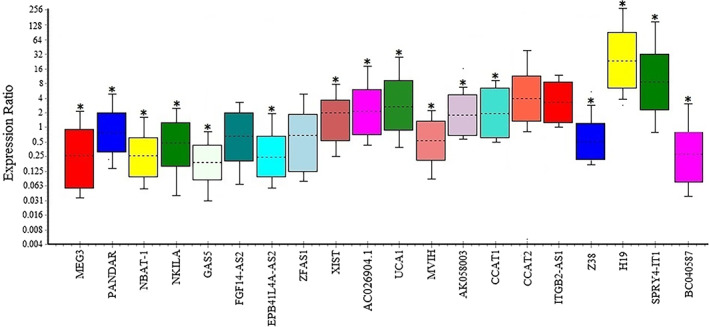
Box‐plot diagrams of the relative lncRNA expression levels in 20 blood samples of BC patients are illustrated over the median of the healthy samples. The fold changes of the analyzed RT‐polymerase chain reaction by REST software (*p < .05)

**FIGURE 2:**
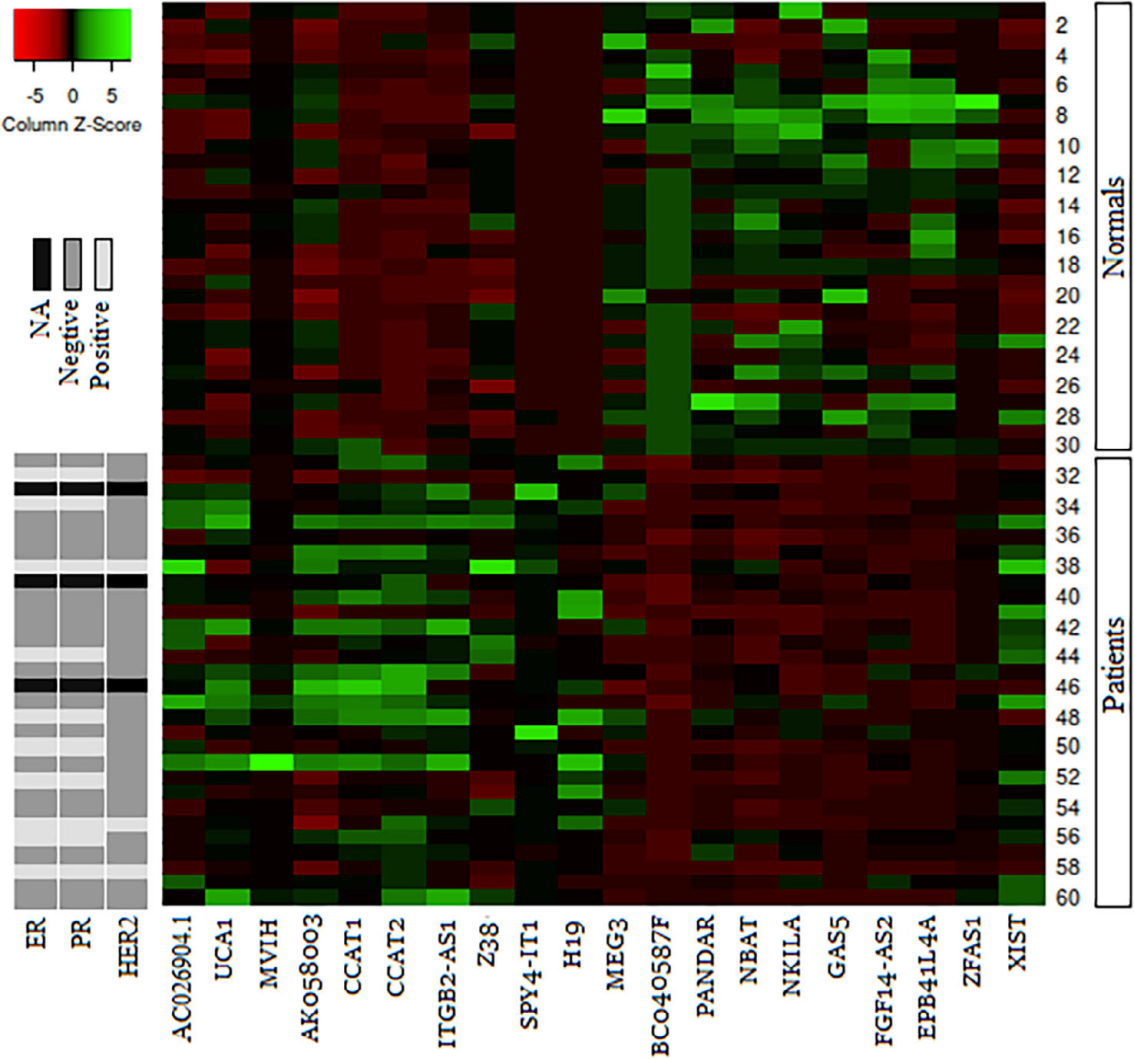
The Heatmap depicts lncRNAs expression levels (columns) in breast cancer patients compared with healthy normal women (rows). Expression values as 2^−∆CT^ are illustrated

The expression level of 20 lncRNAs in blood samples of breast cancer patients showed in Figure 1 and their expression compared with healthy normal women in Figure 2. The results showed the information on fold change of the down‐regulated lncRNAs in the blood samples of breast cancer patients comparing to the healthy women as (*p* < .05) MEG3 (0.216 ± 0.026), NBAT1 (0.233 ± 0.051), NKILA (0.453 ± 0.087), GAS5 (0.188 ± 0.051), Z38 (0.487 ± 0.113), EPB41L4A‐AS2 (0.256 ± 0.057), BC040587 (0.260 ± 0.038). On the other hand, the fold change of over‐expressed lncRNAs (*p* < .05) are as follows: H19 (25.35 ± 3.152), SPRY4‐IT1 (9.062 ± 1.076), CCAT2 (3.12 ± 1.05), ITGB (2.95 ± 0.32), UCA1 (2.817 ± 0.461), AC026904.1 (2.171 ± 0.359), CCAT1 (1.548 ± 0.096), XIST (1.450 ± 0.229), and AK058003 (1.455 ± 0.1; Table 3).

**TABLE 3:** Current and previous reports of the circulating lncRNAs levels in breast cancer

LncRNAs	Expression levels in			
	Tumor tissue	Blood sample	Blood sample (this study)	*p*‐Value
H19	Up‐regulated^38^	Up‐regulated^39^	Up‐regulated (25.350 ± 3.152)	.00
XIST	Down‐regulated^23^	Up‐regulated^40^	Up‐regulated (1.450 ± 0.229)	.023
GAS5	Down‐regulated^16^	Down‐regulated^41^	Down‐regulated (0.188 ± 0.051)	.00
PANDAR	Up‐regulated^42^	No report	No significant change	.487
CCAT1	Up‐regulated^10^, ^11^	No report	Up‐regulated (1.548 ± 0.096)	.001
CCAT2	Up‐regulated^15^	No report	Up‐regulated (3.12 ± 1.05)	.05
UCA1	Up‐regulated^43^	No report	Up‐regulated (2.817 ± 0.461)	.001
BC040587	Down‐regulated^22^	No report	Down‐regulated (0.26 ± 0.038)	.00
SPRY4‐IT1	Up‐regulated^44^	Down‐regulated^39^	Up‐regulated (9.062 ± 1.07)	.00
NBAT1	Down‐regulated^20^	No report	Down‐regulated (0.233 ± 0.051)	.00
AK058003	Up‐regulated^26^, ^45^	No report	Up‐regulated (1.455 ± 0.1)	.011
Z38	Up‐regulated^9^	No report	Down‐regulated (0.487 ± 0.113)	.011
FGF14‐AS2	Down‐regulated^21^	No report	No significant change	.288
MVIH	Up‐regulated^12^	No report	No significant change	.314
EPB41L4A‐AS2	Down‐regulated^25^	No report	Down‐regulated (0.256 ± 0.057)	.00
NKILA	Down‐regulated^34^	No report	Down‐regulated (0.453 ± 0.087)	.009
ZFAS1	Down‐regulated^35^, ^46^	No report	No significant change	.063
AC026904.1	Up‐regulated^38^	No report	Up‐regulated (2.171 ± 0.359)	.021
MEG3	Down‐regulated^44^	Down‐regulated^42^	Down‐regulated (0.216 ± 0.026)	.00
ITGB2‐AS1	Up‐regulated^3^	No report	Up‐regulated (2.95 ± 0.32)	.036

**FIGURE 3:**
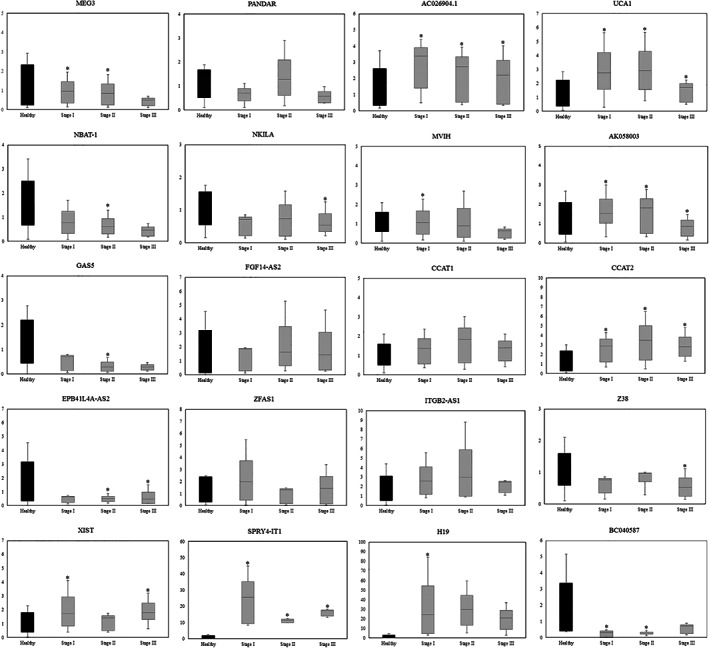
Relative expression levels of a selected lncRNAs in the blood of 30 patients during different stages of breast cancer and 30 healthy controls, which assessed by real‐time polymerase chain reaction. *p*‐Value of lncRNA expression of each stage was determined using the one‐way analysis (ANOVA) followed by Bonferroni post hoc test, and the results compared to healthy individuals (*p < .05). Stage I (*n* = 14), stage II (*n* = 7), stage III (*n* = 9), healthy (*n* = 30)

Expression levels of circulating lncRNAs during different stages of breast cancer were shown in Figure 3, and the lncRNA expression of each stage was compared to healthy individuals using the ANOVA followed by Bonferroni post hoc test.

Assessments of lncRNAs profiles association with clinicopathological features of breast cancer patients showed that the expression level of NKILA, and NBAT1 lncRNAs were related to the tumor size. However, BC040587expression level was related to age, node metastasis, tumor size, and grade of breast cancer patients, and NBAT1 lncRNA expression was also correlated with the patients' age (*p* < .05). Furthermore, there is a statistical association between SPRY4‐IT1 (*p* = .03) and H19 (*p* = .04) expression levels and HER‐2 in cancer patients' blood samples. The correlation between other lncRNAs expression levels and clinicopathological features was not significant (*p* > .05; Table 4).

**TABLE 4:** The relation between clinopathological traits and expression levels of lncRNAs determined by x2 test. The significant relation were shown by *p* < .05

LncRNAs	Clinopathological traits									Statistical parameters
	Age	Tumor size	Grade	Lymph node metastasis	Stage	Histological type	ER	PR	HER2	
H19	0.242	0.294	0.375	0.389	0.375	0.372	0.333	0.333	0.04*	*p*
	2.88	1.62	36.00	18.000	36.000	22.020	8.000	8.000	21.69	X2
XIST	0.362	0.198	0.482	0.305	0.587	0.220	0.382	0.382	0.382	*p*
	5.38	3.33	47.781	26.998	45.226	29.000	16.00	16.000	16.000	X2
GAS5	0.242	0.327	0.635	0.372	0.590	0.234	0.252	0.252	0.565	*p*
	4.338	2.43	32.522	19.33	33.452	21.964	14.811	14.811	10.578	X2
PANDAR	0.1	0.081	0.204	0.319	0.418	0.788	0.397	0.397	0.832	*p*
	4.7	2.78	44.933	21.33	39.147	13.929	12.621	12.621	7.367	X2
CCAT1	0.361	0.505	0.333	0.581	0.278	0.260	0.383	0.383	0.249	*p*
	5.39	3.11	51.667	21.969	53.278	28.00	13.867	13.867	16.000	X2
CCAT2	0.419	0.544	0.433	0.289	0.262	0.476	0.383	0.383	0.249	*p*
	4.25	2.37	40.886	22.995	45.260	19.71	13.867	13.867	16.000	X2
UCA1	0.232	0.219	0.450	0.461	0.318	0.746	0.382	0.382	0.382	*p*
	4.58	3.17	46.538	22.997	50.000	18.207	16.00	16.000	16.000	X2
BC040587	0.05*	0.02*	0.04*	0.024*	0.289	0.150	0.253	0.253	0.253	*p*
	17.000	35.24	78.125	32.878	27.342	17.000	9.000	9.000	9.000	X2
SPRY4‐IT1	0.306	0.647	0.390	0.69	0.283	0.224	0.306	0.306	0.03*	*p*
	1.17	6.000	21.125	7.367	23.111	13.000	6.000	6.000	24.65	X2
NBAT1	0.04*	0.02*	0.664	0.383	0.555	0.462	0.557	0.557	0.256	*p*
	19.35	34.65	39.509	23.33	42.063	21.964	12.621	12.621	17.000	X2
AK058003	0.358	0.559	0.497	0.459	0.267	0.540	0.460	0.460	0.313	*p*
	4.72	2.69	41.4	20.992	47.250	19.71	13.867	13.867	16.000	X2
Z38	0.316	0.25	0.202	0.585	0.210	0.948	0.201	0.201	0.299	*p*
	2.99	2.049	38.393	14.177	38.163	8.016	12.222	12.222	10.673	X2
FGF14‐AS2	0.39	0.193	0.495	0.333	0.705	0.980	0.391	0.391	0.256	*p*
	5.37	3.200	45.446	25.33	40.397	11.250	14.811	14.811	17.000	X2
MVIH	0.276	0.786	0.628	0.500	0.416	0.252	0.537	0.537	0.513	*p*
	4.57	2.45	40.362	21.333	45.333	26.000	11.891	11.891	12.183	X2
EPB41L4A‐AS2	0.47	0.21	0.490	0.281	0.688	0.462	0.454	0.454	0.150	*p*
	5.07	3.05	43.571	25.33	38.929	21.964	11.891	11.891	17.000	X2
NKILA	0.47	0.05*	0.423	0.323	0.423	0.385	0.391	0.391	0.256	*p*
	4.62	29.944	41.083	22.325	41.086	21.213	14.811	14.811	17.000	X2
ZFAS1	0.459	0.306	0.277	0.417	0.640	0.591	0.553	0.553	0.182	*p*
	4.223	2.71	44.800	20.667	36.241	17.949	9.750	9.750	15.000	X2
AC026904.1	0.259	0.232	0.717	0.459	0.500	0.171	0.191	0.191	0.191	*p*
	4.59	2.89	36.343	20.992	41.330	27.000	16.00	16.000	16.000	X2
MEG3	0.353	0.248	0.292	0.578	0.282	0.264	0.382	0.382	0.382	*p*
	5.87	3.42	54.962	22.993	55.274	29.000	16.00	16.000	16.000	X2
ITGB2‐AS1	0.053	0.314	0.696	0.571	0.482	0.882	0.233	0.233	0.233	*p*
	3.47	2.13	29.33	15.33	33.700	10.476	14.00	14.000	14.000	X2

* In case of *p* < .05.

### 3.3 Diagnostic accuracy of circulating lncRNAs

**FIGURE 4:**
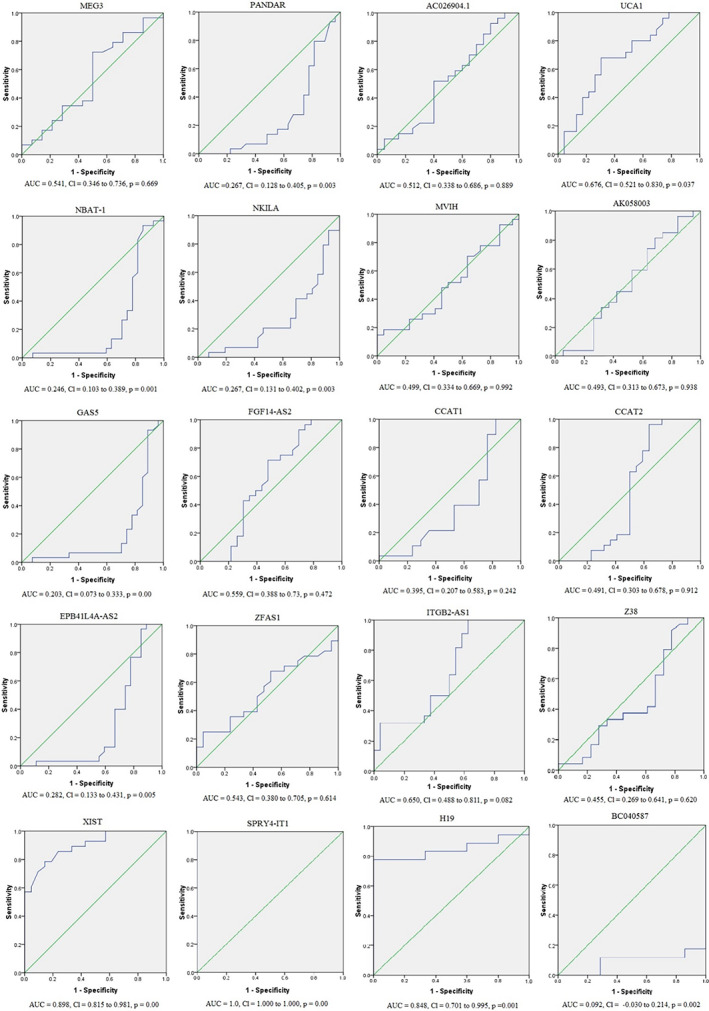
ROC curves of circulating H19, CCAT1, CCAT2, UCA1, SPRY4‐IT1, AK058003, Z38, MVIH, XIST, PANDAR, GAS5, ITGB2‐AS1, MEG3, AC026904.1, ZFAS1, NKILA, EPB41L4A‐AS2, FGF14‐AS2, NBAT1, BC040587 in blood samples of breast cancer patients (*n* = 30) and healthy females (*n* = 30). AUC, area under the curve; ROC, receiver operating characteristic curve

The results of ROC curve analysis illustrated that the blood levels of SPRY4‐IT1, XIST, and H19 lncRNAs have excellent potential in discriminating breast cancer from the healthy controls, showing an AUC of 1.0 (95% CI 1.0–1.0, *p* = .00), 0.898 (95% CI 0.815–0.981, *p* = .00), and 0.848 (95% CI 0.701–0.995, *p* = .01), respectively (Figure 4).

### 3.4 Correlation analysis between the levels of circulating lncRNAs in the breast cancer patients

**FIGURE 5:**
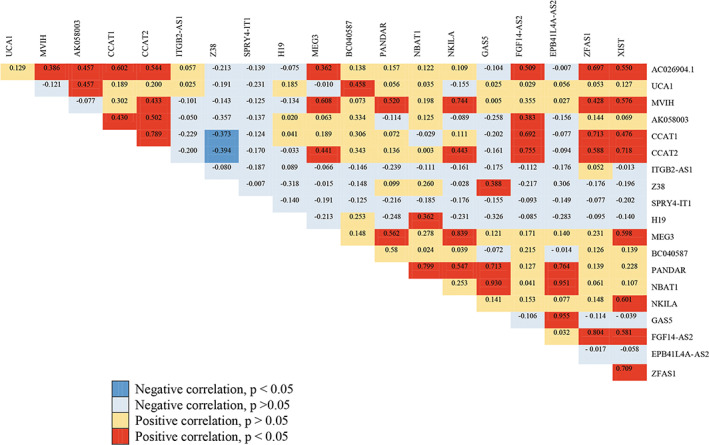
Correlation between the circulating levels of 20 lncRNAs were analyzed by Pearson correlation in the breast cancer patients

According to the Pearson correlation analysis (Figure 5), all the circulating levels of lncRNAs in red color were discovered to be positively related to each other in breast cancer patients, except from CCAT1 and CCAT2 that has a negative relation with Z38.

## 4 DISCUSSION

Identifying highly sensitive and specific lncRNAs for the early diagnosis and prognosis of breast cancer invasion and metastasis remain a hard task. Many studies have explored biomarkers in tumor biopsies, suggesting many candidate RNAs and proteins as biomarkers in various cancers.

The detection of lncRNAs in body fluids, such as blood or urine, could be considered non‐invasive cancer biomarkers. Furthermore, the lncRNAs biomarker characterization could be beneficial for early detection and treatment of breast cancer.^34^ Increasing evidence represented the association of lncRNAs expressed in tumor tissues with cancer development or metastasis.^7^ At the same time, there are few reports of circulating lncRNAs in blood samples of cancer patients as shown in Table 3. We compare the expression levels of 20 lncRNAs in blood samples of breast cancer patients and healthy individuals, and then the correlation between lncRNAs deregulation and clinical characteristics was analyzed in this study. Next, we investigated the sensitivity, specificity, and the potential of circulating lncRNAs in discriminating breast cancer from the healthy controls. Finally, we study the correlation between the levels of different lncRNAs in breast cancer patients.

Considering the results of current study (Table 3), the expression levels of some lncRNAs in patients' blood and cancer tissue were different. The possible explanation for this phenomenon might be the different disease stages among various studies. Besides, the low circulating lncRNA levels compared to lncRNA levels in tissue specimens might be due to both the technical and biological determinants that impact circulating lncRNA levels.^39^, ^47^

According to the results, the blood levels of SPRY4‐IT1, XIST, and H19 lncRNAs have excellent potential in discriminating breast cancer from the healthy controls (Figure 4). There is an association between SPRY4‐IT1, and H19 deregulation and clinical characteristics (Table 4). On the other hand, the dramatic rise in the circulating SPRY4‐IT1, and H19 LncRNAs expression levels are shown in breast cancer patients compared to healthy individuals. Therefore, we suggest SPRY4‐IT1, and H19 lncRNAs can be used as screening biomarkers for early detection of breast cancer.

Our findings approved the results of Jiao et al, who investigated the H19 expression levels in the plasma of breast cancer patients compared with healthy controls.^39^ Dugimont and Adriaenssens illustrated a correlation between H19 expression levels and pathological features such as lymph node metastasis, tumor grades, and the presence of estrogen and progesterone receptors that did not validate in the current research.^48^, ^49^, ^50^ On the other hand, we have found a positive correlation between H19 LncRNA expression level and HER‐2 that indicated H19 as a potential regulator of proliferation in the HER2 enriched subtype (Table 4). In addition, there is a positive correlation between the levels of H19 and NBAT1 lncRNAs in breast cancer blood samples (Figure 5), and the possible association among their functions remain to be confirmed in further studies.

Several studies demonstrated that SPRY4‐IT1 promotes cell growth, invasion and inhibits apoptosis in several types of cancer, including breast cancer.^13^ This statement confirms our finding that the expression levels of SPRY4‐IT1 were significantly overexpressed in blood samples of breast cancer patients in comparison to healthy individuals (Figure 1). In contrast, Jiao et al showed down‐regulation of lncRNA SPRY4‐IT1 in breast cancer patients' plasma compared to healthy female controls. They used different primer pairs for SPRY4‐IT1 amplification, and there is no information about patients' pathological traits. According to Xiang et al, there is no significant relation between SPRY4‐IT1 expression in BC tissues and *molecular* subtypes (ER/PR/HER2) of breast cancer^42^; however, we found a significant relation between SPRY4‐IT1 and HER‐2. Moreover, there is not a significant correlation between SPRY4‐IT1 and other circulating lncRNAs levels in blood samples of breast cancer patients (Figure 5).

Because of strongly associated BC040587 expression with tumor size, grade, and node (Table 4), we suggest the application of BC040587 lncRNA as a diagnostic and prognostic indicator to assess tumor progression that could be prove in larger patients' cohorts.

In conclusion, there is an essential need to search for novel prognostic biomarkers with high specificity and sensitivity for breast cancer screening. In recent years, lncRNAs have been implicated as having oncogenic and tumor suppressor roles and can be used to develop as biomarkers and prognosis factors. In the present study, the dramatic rise in the circulating SPRY4‐IT1, and H19 LncRNAs expression levels are shown in breast cancer patients compared to healthy individuals. Furthermore, because of the significant correlation of H19, and SPRY4‐IT1 lncRNAs with clinicopathological traits and their excellent power in discriminating breast cancer from healthy individuals, they could be considered as the prognostic biomarkers and novel therapeutic targets in breast cancer. We also suggest the possible use of BC040587 lncRNA as a prognostic indicator of breast cancer in case of verification in larger patients' cohorts.

## AUTHOR CONTRIBUTIONS

**Zahra Pourramezan:** Conceptualization (equal); formal analysis (equal); investigation (equal); writing – original draft (equal); writing – review and editing (equal). **Fatemeh Akhavan Attar:** Data curation (equal); investigation (equal). **Maryam Yousefpour:** Data curation (equal); investigation (equal). **Masomeh Azizi:** Formal analysis (equal); supervision (equal); validation (equal). **mana oloomi:** Conceptualization (equal); methodology (equal); project administration (equal); supervision (equal).

## FUNDING INFORMATION

This work was supported by the Pasteur Institutes of Iran [grant number 1046].

## CONFLICT OF INTEREST

The authors declare no conflict of interest.

## ETHICS STATEMENT

The study protocols were approved by the institutional ethics review board at Pasteur Institute of Iran (IR.PII.REC.1397.008). Informed written consent was taken from every patient before recruiting in the study. This study has been done in accordance to the guidelines of the *Cancer Reports* journal, and has been performed in an ethical and responsible way, with no research misconduct, which includes, but is not limited to data fabrication and falsification, image manipulation, plagiarism, biased reporting, unethical research, redundant or duplicate publication, authorship abuse, and undeclared conflicts of interest.

## Data Availability

Data access will be granted to anonymized patient‐level data, protocols, and clinical study reports after approval by an independent scientific review panel.

## References

[1] Harbeck N , Penault‐Llorca F , Cortes J , et al. Breast cancer. Nat Rev Dis Primers. 2019;5:66. doi:10.1038/s41572-019-0111-2 31548545

[2] Zendehdel K . Cancer statistics in I.R. Iran in 2018. Basic Clin Cancer Res. 2019;11(1):1‐4. doi:10.18502/bccr.v11i1.1645

[3] Liu M , Gou L , Xia J , et al. LncRNA ITGB2‐AS1 could promote the migration and invasion of breast cancer cells through up‐regulating ITGB2. Int J Mol Sci. 2018;19:1866. doi:10.3390/ijms19071866 29941860 PMC6073814

[4] Oloomi M , Bouzari S , Mohagheghi MA , Khodayaran‐Tehrani H . Molecular markers in peripheral Bloodof Iranian women with breast cancer. Cancer Microenviron. 2013;6:109‐116. doi:10.1007/s12307-012-0118-7 22828927 PMC3601212

[5] Wu ZJ , Li Y , Wu YZ , et al. Long non‐coding RNA CCAT2 promotes the breast cancer growth and metastasis by regulating TGF‐beta signaling pathway. Eur Rev Med Pharmacol Sci. 2017;21:706‐714. 28272713

[6] Xi J , Feng J , Li Q , Li X , Zeng S . The long non‐coding RNA lncFOXO1 suppresses growth of human breast cancer cells through association with BAP1. Int J Oncol. 2017;50:1663‐1670. doi:10.3892/ijo.2017.3933 28339037

[7] Zhang M , Wu WB , Wang ZW , Wang XH . lncRNA NEAT1 is closely related with progression of breast cancer via promoting proliferation and EMT. Eur Rev Med Pharmacol. 2017;21:1020‐1026. 28338194

[8] Shi T , Gao G , Cao Y . Long noncoding RNAs as novel biomarkers have a promising future in cancer diagnostics. Dis Markers. 2016;2016:1‐10. doi:10.1155/2016/9085195 PMC4842029 27143813

[9] Deng R , Liu B , Wang Y , et al. High expression of the newly found long noncoding RNA Z38 promotes cell proliferation and oncogenic activity in breast cancer. J Cancer. 2016;7(5):576‐586. doi:10.7150/jca.13117 27053956 PMC4820734

[10] Zhang ZF , Liu T , Li Y , Li S . Overexpression of long non‐coding RNA CCAT1 is a novel biomarker of poor prognosis in patients with breast cancer. Int J Clin Exp Pathol. 2015;8(8):9440‐9445. 26464701 PMC4583933

[11] Xin Y , Li Z , Shen J , Chan MT , Wu WKK . CCAT1: a pivotal oncogenic long non‐coding RNA in human cancers. Cell Prolif. 2016;49:255‐260. doi:10.1111/cpr.12252 27134049 PMC6496795

[12] Lei B , Xu SP , Liang XS , et al. Long non‐coding RNA MVIH is associated with poor prognosis and malignant biological behavior in breast cancer. Tumor Biol. 2016;37:5257‐5264. doi:10.1007/s13277-015-4360-8 26555546

[13] Shi Y , Li J , Liu Y , et al. The long noncoding RNA SPRY4‐IT1 increases the proliferation of human breast cancer cells by upregulating ZNF703 expression. Mol Cancer. 2015;14:51. doi:10.1186/s12943-015-0318-0 25742952 PMC4350857

[14] Huang J , Zhou N , Watabe K , et al. Long non‐coding RNA UCA1 promotes breast tumor growth by suppression of p27 (Kip1). Cell Death Dis. 2014;5:e1008. doi:10.1038/cddis.2013.541 24457952 PMC4040676

[15] Cai Y , He J , Zhang D . Long noncoding RNA*CCAT2* promotes breast tumor growth by regulating the Wnt signaling pathway. Onco Targets Ther. 2015;8:2657‐2664. doi:10.2147/OTT.S90485 26442763 PMC4590572

[16] Li S , Zhou J , Wang Z , Wang P , Gao X , Wang Y . Long noncoding RNA GAS5 suppresses triple negative breast cancer progression through inhibition of proliferation and invasion by competitively binding miR‐196a‐5p. Biomed Pharmacother. 2018;104:451‐457. doi:10.1016/j.biopha.2018.05.056 29793177

[17] Yang Z , Sun Y , Liu R , Shi Y , Ding S . Plasma long noncoding RNAs PANDAR, FOXD2‐AS1, and SMARCC2 as potential novel diagnostic biomarkers for gastric cancer. Cancer Manag Res. 2019;11:6175‐6184. doi:10.2147/CMAR.S201935 31308753 PMC6613614

[18] Collette J , Bourhis XL , Adriaenssens E . Regulation of human breast cancer by the long non‐coding RNA H19. Int J Mol Sci. 2017;18:2319. doi:10.3390/ijms18112319 29099749 PMC5713288

[19] Jiang S , Chen R , Yu J , et al. Clinical significance and role of LKB1 in gastric cancer. Mol Med Rep. 2016;13:249‐256. 26549522 10.3892/mmr.2015.4508

[20] Hu P , Chu J , Wu Y , et al. NBAT1 suppresses breast cancer metastasis by regulating DKK1 via PRC2. Oncotarget. 2015;6(32):32410‐32425. doi:10.18632/oncotarget.5609 26378045 PMC4741702

[21] Yang F , Liu YH , Dong SY , et al. A novel long non‐coding RNA FGF14‐AS2 is correlated with progression and prognosis in breast cancer. Biochem Biophys Res Commun. 2016;470:479‐483. doi:10.1016/j.bbrc.2016.01.147 26820525

[22] Yayun C , Huang S , Yuan L , et al. Role of BC040587 as a predictor of poor outcome in breast cancer. Cancer Cell Int. 2014;14:123. doi:10.1186/s12935-014-0123-7 25435812 PMC4246536

[23] Zheng R , Lin S , Guan L , et al. Long non‐coding RNA XIST inhibited breast cancer cell growth, migration, and invasion via miR‐155/CDX1 axis. Biochem Biophys Res Commun. 2018;498(4):1002‐1008. doi:10.1016/j.bbrc.2018.03.104 29550489

[24] Liu B , Sun L , Liu Q , et al. A cytoplasmic NF‐kappaB interacting long noncoding RNA blocks IkappaB phosphorylation and suppresses breast cancer metastasis. Cancer Cell. 2015;27:370‐381. doi:10.1016/j.ccell.2015.02.004 25759022

[25] Xu S , Wang P , You Z , et al. The long non‐coding RNA *EPB41L4A‐AS2* inhibits tumor proliferation and is associated with favorable prognoses in breast cancer and other solid tumors. Oncotarget. 2016;7(15):20704‐20717. doi:10.18632/oncotarget.8007 26980733 PMC4991486

[26] He X , Zheng Y , Zhang Y , et al. Long non‐coding RNA AK058003, as a precursor of miR‐15a, interacts with HuR to inhibit the expression of γ‐synuclein in hepatocellular carcinoma cells. Oncotarget. 2017;8(6):9451‐9465. doi:10.18632/oncotarget.14276 28035067 PMC5354744

[27] Aagaard K , Petrosino J , Keitel WM , et al. The human microbiome project strategy for comprehensive sampling of the human microbiome and why it matters. FASEB J. 2013;27:1012‐1022. doi:10.1096/fj.12-220806 23165986 PMC3574278

[28] Zhang Y , Zhu Z , Huang S , et al. LncRNA XIST regulates proliferation and migration of hepatocellular carcinoma cells by acting as miR‐497‐5p molecular sponge and targeting PDCD4. Cancer Cell Int. 2019;19:198. doi:10.1186/s12935-019-0909-8 31384173 PMC6664491

[29] Lai Y , Chen Y , Lin Y , Ye L . Down‐regulation of LncRNA CCAT1 enhances radiosensitivity via regulating miR‐148b in breast cancer. Cell Biol Int. 2018;42:227‐236. doi:10.1002/cbin.10890 29024383

[30] Gu L , Lu LS , Zhou DL , Liu ZC . UCA1 promotes cell proliferation and invasion of gastric cancer by targeting CREB1 sponging to miR‐590‐3p. Cancer Med. 2018;7(4):1253‐1263. 29516678 10.1002/cam4.1310 PMC5911610

[31] Ye Y , Gu J , Liu P , et al. Long non‐coding RNA SPRY4‐IT1 reverses cisplatin resistance by downregulating MPZL‐1 via suppressing EMT in NSCLC. OncoTargets Ther. 2010;13:2783‐2793. doi:10.2147/OTT.S232769 PMC7135170 32308413

[32] Nie ZL , Wang YS , Mei YP , et al. Prognostic significance of long noncoding RNA Z38 as a candidate biomarker in breast cancer. J Clin Lab Anal. 2018;32(1):e22193. doi:10.1002/jcla.22193 28247935 PMC6816943

[33] Wang A , Du L , Jiang K , Kong Q , Zhang X , Li L . Long noncoding RNA microvascular invasion in hepatocellular carcinoma is an indicator of poor prognosis and a potential therapeutic target in gastric cancer. J Cancer Res Ther. 2019;15(1):126‐131. doi:10.4103/0973-1482.204882 30880767

[34] Wu W , Chen F , Cui X , et al. LncRNA NKILA suppresses TGF‐b‐induced epithelial–mesenchymal transition by blocking NF‐jB signaling in breast cancer. Int J Cancer. 2018;143:1‐12. doi:10.1002/ijc.31605 29761481

[35] Fan S , Fan C , Liu N , Huang K , Fang X , Wang K . Downregulation of the long non‐coding RNA ZFAS1 is associated with cell proliferation, migration and invasion in breast cancer. Mol Med Rep. 2018;17:6405‐6412. doi:10.3892/mmr.2018.8707 29532866 PMC5928617

[36] Dai J , Xu LJ , Han GD , et al. Down‐regulation of long non‐coding RNA ITGB2‐AS1 inhibits osteosarcoma proliferation and metastasis by repressing Wnt/β‐catenin signaling and predicts favourable prognosis. Artif Cell Nanomed Biotechnol. 2018;46(suppl 3):S783‐S790. doi:10.1080/21691401.2018.1511576 30260245

[37] Unal I . Defining an optimal cut‐point value in ROC analysis: an alternative approach, computational and mathematical Methods in medicine. Comput Math Methods Med. 2017;2017:1‐14. doi:10.1155/2017/3762651 PMC5470053 28642804

[38] Lottin S , Adriaenssens E , Dupressoir T , et al. Overexpression of an ectopic H19 gene enhances the tumorigenic properties of breast cancer cells. Carcinogenesis. 2002;23:1885‐1895. doi:10.1093/carcin/23.11.1885 12419837

[39] Jiao ZY , Tian Q , Li N , Wang HB , Li KZ . Plasma long non‐coding RNAs (lncRNAs) serve as potential biomarkers for predicting breast cancer. Eur Rev Med Pharmacol Sci. 2018;22(7):1994‐1999. 29687854 10.26355/eurrev_201804_14727

[40] Salama EA , Adbeltawab RE , El Tayebi HM . XIST and TSIX: novel cancer immune biomarkers in PD‐L1‐overexpressing breast cancer patients. Front Oncol. 2010;9:1‐13. doi:10.3389/fonc.2019.01459 PMC6966712 31998636

[41] Han L , Ma P , Liu SM , Zhou X . Circulating long noncoding RNA GAS5 as a potential biomarker in breast cancer for assessing the surgical effects. Tumor Biol. 2016;37(5):6847‐6854. doi:10.1007/s13277-015-4568-7 26662314

[42] Ali MA , Shaker OG , Alazrak M , et al. Association analyses of a genetic variant in long non‐coding RNA MEG3 with breast cancer susceptibility and serum MEG3 expression level in the Egyptian population. Cancer Biomark. 2020;28:1‐5. doi:10.3233/CBM-191072 32176630 PMC12662337

[43] Tuo YL , Li XM , Luo J . Long noncoding RNA UCA1 modulates breast cancer cell growth and apoptosis through decreasing tumor suppressive miR‐143. Eur Rev Med Pharmacol Sci. 2015;19:3403‐3411. 26439035

[44] Sun L , Li Y , Yang B . Downregulated long noncoding RNA MEG3 in breast cancer regulates proliferation, migration and invasion by depending on p53's transcriptional activity. Biochem Biophys Res Commun. 2016;478:323‐329. doi:10.1016/j.bbrc.2016.05.031 27166155

[45] He K , Wang P . Unregulated long non‐coding RNA‐AK058003 promotes the proliferation, invasion and metastasis of breast cancer by regulating the expression levels of the gamma‐synuclein gene. Exp Ther Med. 2015;9:1727‐1732. doi:10.3892/etm.2015.2323 26136884 PMC4471698

[46] Askarian‐Amiri ME , Crawford J , French JD , et al. SNORD‐host RNA Zfas1 is a regulator of mammary development and a potential marker for breast cancer. RNA. 2011;17:878‐891. doi:10.1261/rna.2528811 21460236 PMC3078737

[47] Schlosser K , Hanson J , Villeneuve PJ , et al. Assessment of circulating LncRNAs under physiologic and pathologic conditions in humans reveals potential limitations as biomarkers. Sci Rep. 2016;6:36596. doi:10.1038/srep36596 27857151 PMC5114641

[48] Dugimont T , Adriaenssens E . H19 mRNA‐like noncoding RNA promotes breast cancer cell proliferation through positive control by E2F1. J Biol Chem. 2005;280:29625‐29636. 15985428 10.1074/jbc.M504033200

[49] Sang Y , Tang J , Li S , et al. LncRNA PANDAR regulates the G1/S transition of breast cancer cells by suppressing p16^INK4A^ expression. Sci Rep. 2016;6:22366. doi:10.1038/srep22366 26927017 PMC4772134

[50] Li GY , Wang W , Sun JY , et al. Long non‐coding RNAs AC026904.1 and UCA1: a “one‐two punch” for TGF‐β‐induced SNAI2 activation and epithelial‐mesenchymal transition in breast cancer. Theranostics. 2018;8(10):2846‐2861. doi:10.7150/thno.23463 29774079 PMC5957013

